# Railway contactless checkout process with identification assisted by gait recognition

**DOI:** 10.1038/s41598-024-64637-w

**Published:** 2024-06-17

**Authors:** Beibei Li, Jiansheng Zhu, Wen Li

**Affiliations:** 1 Institute of Computing Technologies, China Academy of Railway Sciences Group Co, Ltd., Daliushu Road 2, Beijing, 100081 China; 2 Department of Science, Technology and Information, China Railway Co, Ltd., Fuxing Road 10, Beijing, 100844 China; 3 Passenger Transport Department, China Railway Travel Technology Co, Ltd., Daliushu Road 2, Beijing, 100081 China

**Keywords:** Gait recognition, Contactless checkout, Identification, Dwsegnet, GaitSet, Engineering, Electrical and electronic engineering

## Abstract

With business process optimization, technological advancement, equipment capability enhancement, and other means, the Railway Passenger Service Department in China is consistently working to improve the efficiency and convenience of passenger entry and exit procedures at railway stations. Concerning passengers’ checkout, not only conventional identification approaches based on manual control, identification card, and magnetic thermal paper ticket are supported, but also a recent contactless identification process based on face recognition is applied in some stations. To further improve the contactless identification ability for checkout, an advanced contactless checkout process based on gait-augmented face recognition is innovatively put forward, in which a weakly-supervised body segmentation network named Dwsegnet and an improved GaitSet model are proposed. Through comparison with various models, the effectiveness of both Dwsegnet and the improved GaitSet is validated. Specifically, the contactless identification rate of gait-augmented face recognition is improved by 2.31% when compared to single-modal face recognition, which demonstrates the superiority of the proposed process.

## Introduction

With the pioneer application of railway e-ticket in 2018 and its subsequent popularization among railway stations in China, the identification process of checkout for passengers was supported by not only the conventional approaches based on manual checking, magnetic thermal paper ticket, and identification card, but also other means based on QR code and specific media such as China Railway Yintong card. With the aid of face recognition technology, the novel face-based checkout procedures like “sense-free checkout” at Taizicheng station and “contact-free checkout” at Shanwei station have been implemented and researched in an effort to continuously improve the convenience of checkout for railway passengers. This has increased checkout efficiency and boosted the caliber of service rendered by station management personnel. However, due to the demand for warmth in cold areas and protection from the COVID-19 epidemic, it is common for passengers to wear masks, scarves, or other covers on their faces, which challenges face recognition a lot and degrades the effectiveness of the cooperative face recognition method to some extent for requiring passengers to remove the cover.

Considering multiple characteristics such as contact-free, less cooperative, data collectible at long distances, and a mature biometric method, this research delves deeper into a novel contactless checkout process that employs gait recognition as a supplement to face recognition for continuously improving the checkout convenience for passengers. Compared to other biometric recognition methods, with advantages of recognition at long distances and anti-camouflage, gait recognition has been tentatively applied in various scenarios such as clinical medicine^1^, sport science^2^, and population analysis^3^ for it enables constructing serialized unique gait features tailored to the human silhouettes to recognize and classify the target.

Typical gait recognition algorithms are mainly divided into two categories: the traditional body modeling^4^ and the booming model-free^5^ methods. Considering complicated variations in body posture during the walking trajectory, the body modeling methods aim to establish a reliable gait representation model to extract gait features as much as possible. Bobick and Johnson^6^ presented a gait recognition technique that recovers static body and stride parameters of subjects. Cunado et al.^7^ directly extracted gait signature from the evidence gathering process by using a Fourier series, to describe the motion of the upper leg and apply temporal evidence gathering techniques to extract the moving feature from a sequence of images. Yoo et al.^8^ extracted trajectory-based kinematic features from image sequences by using a 2D stick figure model to represent the human body, and joint angles and angular velocities to describe the gait motion. Qiao et al.^9^ proposed Posegait for real-time human gesture grading. Ghebleh and Moghaddam^10^ proposed a robust scheme by employing a view transformation model (VTM) based on sparse and redundant (SR) representation. Guo^11^ based on Kinect bone information to identify the gait by fusing the improved dynamic time warping (DTW) algorithm and k-nearest-neighbor (KNN) algorithm. Instead of establishing a specific subject model, the model-free methods directly extract gait features and classifies them by analyzing gait, posture, and contour in the gait sequences. The commonly discriminative strategies in model-free methods contain silhouette-based, machine-learning-based, statistics-based, and deep-learning-based methods. The silhouette-based methods mainly include averaged silhouette^12^, gait energy image^13^, dynamics weight mask^14^, dynamic gait energy^15^, pose energy image^16^, and gait entropy image^17^, etc. The deep-learning-based methods mainly include principal component analysis (PCA)^18–21^ and Fourier transform^22,23^ in the early stage, and then cross-applied to many other methods. The statistics-based methods include optical flow distribution^24,25^, probability distribution^26^ and texture distribution^27^. Recently, deep learning is becoming the mainstream technique of model-free gait recognition algorithms, and gradually integrating multi-level block structure^28^, attention mechanisms^29^, and so on.

Experimentally, a variety of factors, such as the quality of collected gait data, data processing techniques, and gait recognition algorithms, will influence how accurate gait recognition is. Building on the using railway checkout process with face recognition, this paper designs a contactless checkout process called “gait-augmented face recognition” with several algorithmic innovations. To validate our methodology, the on-site experiments were carried out with the freshly gathered real-world data from the line of Beijing–Zhangjiakou high-speed railway, in which the gallery set was built with data in Qinghe Station, and the probe set was built with data in Badaling Great Wall Station. According to the experimental outcomes, although the proposed contactless checkout process can marginally raise exit efficiency to a certain extent, it is still unfit for usage in production environment.

## Railway contactless checkout via face recognition

### Business Process

**Figure 1:**
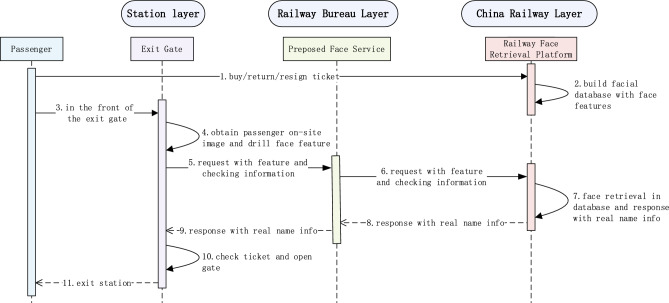
The contactless checkout process based on face recognition.

In the field of railway passenger transportation, the study on 1:N mode face retrieval for contactless checkout^30,31^ has been explored. A railway face retrieval platform has been conducted to support passengers’ senseless identification with an embedded ticket checking process, improving the convenience level as they exit the station. Figure 1 illustrates the face-recognition-based contactless checkout process.

I Face Gallery. When a passenger finishes ticket purchasing, his/her real-name information will be collected. Railway Face Retrieval Platform will then interface with the police system to obtain the passenger’s face and extract facial feature by calling face algorithm service. In order to build a face gallery, which is essential for face retrieval in contactless checkout, facial feature, train code, departure date, real-name information, and railway station details will be associated together in Railway Face Retrieval Platform to be face gallery. Face gallery will be dynamically updated with the change of ticket return or resign operated by the passenger.
II Face Probe. When a passenger arrives at exit gate for checkout, his/her face on the spot is gathered by the integrated camera in gate and then dealt with to extract facial feature through embedded face program in gate, which means that face probe is generated.
III Face Retrieval. To identify the passenger contactlessly, along with face feature and railway station details, the program in gate will call remote service in Railway Face Retrieval Platform and a face retrieval will be performed within the face gallery and face probe. By calculating the similarity among facial features, an ordered score will be outputted. If the highest resemblance exceeds the predetermined threshold, face retrieval succeeds and corresponding real-name information will response. Once the gate receives the successful retrieval response, the ticket will be validated, the door will open, and the closed-loop management of a passenger’s contactless checkout process will finish.

### Key technology of face retrieval

The key technology of contactless checkout is face retrieval, whose underlying technologies include face detection, face alignment and face recognition.

1. Face detection. With RetinaFace^32^, the requirement of pixel-level face detection and positioning of multi-scale faces is realized in the railway face retrieval platform by fusing external supervision with self-supervised multi-task learning. The multi-task loss function $L_{det}$ innovatively proposed by RetinaFace is shown in Formula 1.
  1 $$\begin{aligned} L_{det}=L_{cls}(p_{i},p_{i}^{*})+\lambda _{1}p_{i}^{*}L_{box}(t_{i},t_{i}^{*})+\lambda _{2}p_{i}^{*}L_{pts}(l_{i},l_{i}^{*})+\lambda _{3}p_{i}^{*}L_{pixel} \end{aligned}$$
  where *i* denotes a possible location, *i.e.,* anchor, of faces, $t_{i}$ and $l_{i}$ are offsets of face box and five landmarks, $t_{i}^{*}$ and $l_{i}^{*}$ is the corresponding ground-truth, $p_{i}$ is the predicted probability of anchor *i* being a face, and $p_{i}^{*}$ is 1 for the positive anchor and 0 for the negative anchor. The classification loss $L_{cls}$ is the binary cross entropy loss for binary classes (face/not face). $L_{box}$ is the regression loss of face box, $L_{pts}$ is the regression loss of 5 face landmarks, $L_{pixel}$ is regression loss of dense face landmarks, $\lambda _{1}$, $\lambda _{2}$, and $\lambda _{3}$ are the corresponding loss weight.
2. Face alignment. The research mainly realizes the geometric transformation and spatial normalization based on facial key points.
3. Face recognition. Facial feature is extracted from the aligned face, and face recognition is achieved by measuring the similarity between different face features. The network model in railway face retrieval platform employs the improved ResNet model, and adopts a loss function $L_{rec}$ as shown in Formula 2 for training.
  2 $$\begin{aligned} L_{rec}=-\frac{1}{b}\sum _{i=1}^b log\frac{e^{s\cdot {cos(\theta _{y_i}+m)}}}{e^{s\cdot {cos(\theta _{y_i}+m)}}+\sum _{i=1,j\ne {y_i}}^n e^{s\cdot {cos\theta _{y_i}}}} \end{aligned}$$
  where *b* is the batch size, *s* is proportional scaling value, *n* is number of identities, *m* is margin of the decision boundary, $\theta$ is angle, and $y_i$ is the identity label.

## Railway contactless checkout process assisted by gait recognition

In order to further improve the capability of contactless checkout process for railway passengers, a multi-modal fusion method based on face recognition and gait recognition is researched in this paper.

### Integrated business process

**Figure 2:**
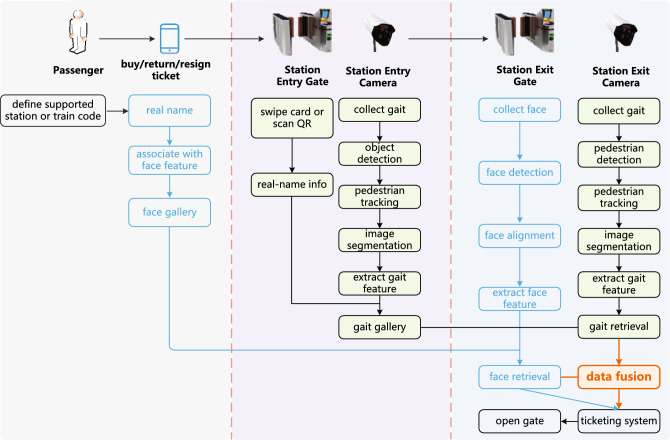
The contactless checkout process assisted by gait recognition.

In order to construct passengers’ gait database, the gait data are gathered as gallery upon passengers check-in, and the gait data are collected as probe set upon passengers check-out. Based on the present contactless checkout process using face recognition with real-name information, the overall business process of multi-modal contactless checkout process is illustrated in Fig. 2.

At the railway station exit, the embedded camera in gate is employed for collecting face images, and the additional video camera is deployed for collecting gait data. The gate’s master controller will perform a random round-robin mode to verify which means—such as examining the magnetic thermal paper ticket, scanning the QR code, or authenticating the face image—is used for check-out when passengers arrive at a specific area ahead of the station gate. In the event that face retrieval fails, the gait feature can be further utilized for gait-augmented face recognition to match the identification through gait retrieval. The master controller uses gait-augmented facial recognition to enhance the efficiency of contactless checkout for railway passenger transportation, and if the identification process succeeds, it will interact with the ticket checking system to indicate the departure status and open the gate door for passenger clearance.

### Optimized algorithm for body segmentation

Generally, after a series of preprocessing of gait data such as pedestrian detection, pedestrian cropping, body segmentation, image binarization, and size standardization, a binary gait sequence with a uniform size forms. In cases of railway passenger service scenarios, this paper optimizes and improves the ability of body segmentation algorithm and gait recognition algorithm.

**Figure 3:**
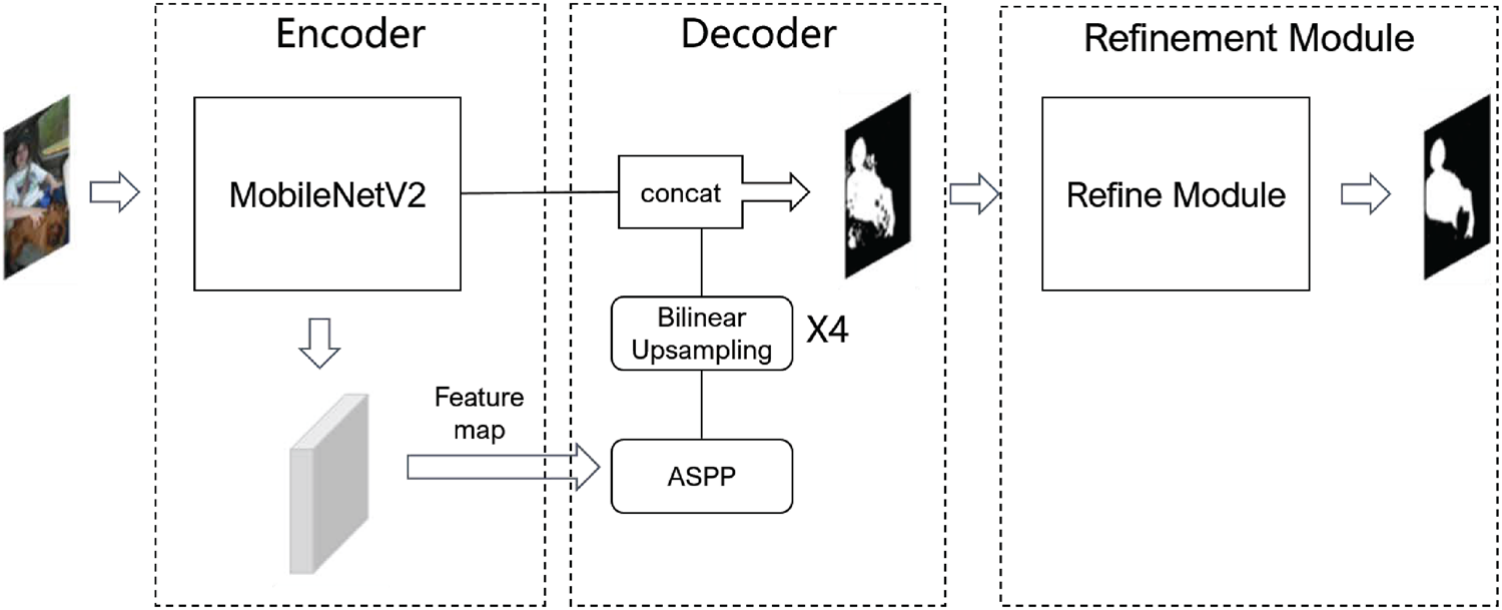
Overall structure of Dwsegnet.

Annotating data is typically costly, time-consuming, and labor-intensive when it comes to body segmentation for gait identification. In recent years, Wei et al.^33^ and Lee et al.^34^ have introduced weakly and semi-supervised semantic body segmentation, which brought fine segmentation results. Both of them construct a Class Activation Map (CAM) with the low-level feature, and it has been proved that the CAM and segmentation map have an inherent link. Motivated by this, the segmentation network can be trained to extract more diversified semantic information, including a knapsack and purse, in addition to capturing pedestrian gait-related aspects by leveraging the low-level feature during transfer learning. In view of this, we propose Dwsegnet, an end-to-end segmentation network model, as shown in Fig. 3. Three components make up the proposed Dwsegnet: an encoder, a decoder, and a refinement module. A hybrid loss function is put forward for training, which integrates global feature and local feature and assigns different weight to yield a body segmentation silhouette with complete and smooth edges.

As to the encoder of the innovative Dwsegnet segmentation network model, MobilenetV2^35^ is adopted as backbone with a bottleneck structure, which cascades a 1 $\times$ 1 convolution layer, a 3 $\times$ 3 depth-wise separable convolution layer, and a 1 $\times$ 1 convolution layer. The first 1 $\times$ 1 convolution layer reduces the number of input channels, the 3 $\times$ 3 depth-wise separable convolution layer realizes lightweight and efficient convolution operation, and the last 1 $\times$ 1 convolution layer is responsible for recovering the number of input channels. To improve computational efficiency while maintaining the accuracy, each part in the bottleneck structure utilizes a linear activation function rather than ReLU. Meanwhile, MobileNetV2 can achieve object detection comparable to the performance of deeper and wider network structure with lower latency and power consumption thanks to the savings in model parameters and computation by incorporating residual structure^36^ into bottleneck structure. This makes MobileNetV2 suitable for deployment in resource-constrained edge devices such as mobile phones or station gates.

In the decoder, the ASPP (Atrous Spatial Pyramid Pooling) module in DeepLabV3 network architecture is introduced to obtain multi-scale semantic segmentation information from different receptive fields through atrous convolutions^37^ and global pooling with different sampling rates. Afterwards, through bilinear interpolation and spatial pyramid pooling with strides, the feature map is up-sampled to the original size. By preventing gradient vanishing and explosion and speeding up convergence during training, the final connection operation enables balancing the deep and shallow features.

Without altering the image size, the refinement module employs a residual structure and symmetric encoder-decoder structure. This part can eliminate edge noise from the prediction map and decrease the inaccuracy of overfitting weakly annotated labels. Subsequently, the output image is then joined to and normalized with the original image, completing and smoothing the final segmentation result.

For the segmentation network model of Dwsegnet, a hybrid loss function $loss_{Hybrid}$ is proposed, as shown in Eqs. (3), (4), and (5).

3 $$\begin{aligned}{} & {} loss_{Hybrid}= l_{SSIM}+l_{Dice} \end{aligned}$$

4 $$\begin{aligned}{} & {} l_{SSIM}=1-\frac{(2\mu _x\mu _y+c_1)(2\sigma _{xy}+c_2)}{(\mu _x^2+\mu _y^2+c_1)(\sigma _x^2+\sigma _y^2+c_2)} \end{aligned}$$

5 $$\begin{aligned}{} & {} l_{Dice}=1-2\sum _{h}^H\sum _w^W\frac{P_{(h,w)}G_{(h,w)}}{\sum _{h}^H\sum _w^W[P_{(h,w)}+G_{(h,w)}]} \end{aligned}$$

$l_{SSIM}$ denotes for structural similarity loss, which expresses the local loss and focuses on structural similarity rather than pixel-level differences. $l_{Dice}$ indicates sample similarity loss, which expresses similarities of global distribution between prediction map and segmentation map. In Eq. (4) and (5), *x* and *y* is image block of prediction box and groundtruth box, $\mu _x$ and $\mu _y$ denote the mean value of *x* and *y*, $\sigma _x$ and $\sigma _y$ denote the variance of *x* and *y*, $\sigma _{xy}$ is the covariance of *x* and *y*, $G_{(h,w)}\epsilon {\{0,1\}}$ is the groundtruth value at coordinate (*h*, *w*), $P_{(h,w)}\epsilon {\{0,1\}}$ is predicted value at coordinate (*h*, *w*). To avoid possible denominators of zero, a small value is added with $c_1=0.01^2$ and $c_2=0.03^2$.

### Improved gait recognition algorithm based on GaitSet

To select a good gait recognition algorithm for our real production environment, a comprehensive survey has been performed. The Rank-k accuracy of 6 baselines are summarized in Table 1 through a comparison with the recent gait recognition baselines trained on the GREW train set and evaluated on test set in the previous literature^38^. Results indicate that GaitSet are superior approaches for gait recognition in the wild, scoring 46.28% in terms of Rank-1 metric, which turns out GaitSet to be our primary option.

**Table 1:** Rank-1, Rank-5, Rank-10, Rank-20 (%) of baselines.

Baseline	Rank-1	Rank-5	Rank-10	Rank-20
GEINet	6.82	13.42	16.97	21.01
TS-CNN	13.55	24.55	30.15	37.01
GaitSet	46.28	63.58	70.26	76.82
GaitPart	44.01	60.68	67.25	73.47
PoseGait	0.23	1.05	2.23	4.28
GaitGraph	1.31	3.46	5.08	7.51

**Figure 4:**
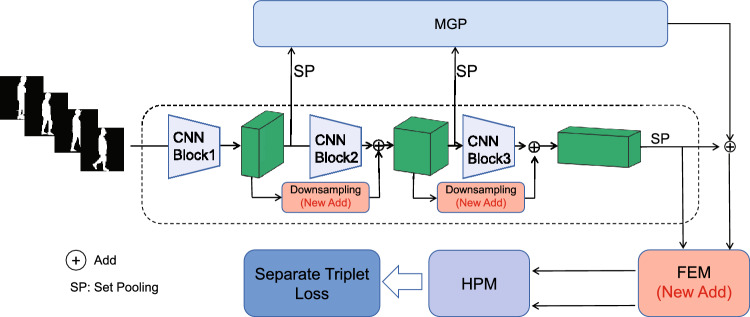
Improved Gaitset network structure.

The study of gait recognition algorithms has advanced significantly in recent years, and under laboratory conditions the recognition accuracy can reach as high as 95%^39^. Nonetheless, the practical implementation of gait recognition poses significant challenges due to various circumstances such as widespread pedestrian occlusion, halting, and others. Considering the factors of gait recognition algorithms such as public accessibility and the flexibility of gait recognition, GaitSet is selected as the baseline to treat gait sequences as an unordered set that is immune to frames permutation^40,41^. In addition, an improved network structure for GaitSet is shown in Fig. 4. Compared to the original GaitSet, the cascading fusion of multi-scale features and enhancement of features further improve gait recognition accuracy.

Multilayer Global Pipeline (MGP) and Horizontal Pyramid Mapping (HPM) are the off-the-shell modules of GaitSet. Block1, Block2, and Block3 represent different blocks of the convolutional neural network, $f_1$,$f_2$, and $f_3$ denote feature maps generated by CNN blocks, SP denotes set pooling. By performing SP operation on gait features, set-level features are obtained. In the improved gait recognition network structure, $f_2$ is obtained by adding downsampling feature $f_1$ with the next-level feature generated by Block 2, and $f_3$ is obtained in the same way, which can be described as Eq. (6).

6 $$\begin{aligned} f_{i+1}=B(f_i)+D(f_i) \end{aligned}$$

where $f_{i+1}$ is the next-level feature of $f_i$, B represents the convolutional block, D portrays the down-sampling operation. The operation of element summation in Eq. (6) can also be replaced by other operations like channel-wise concatenating. Different from other features fusion methods that aggregate features of each stage as the final feature, the proposed multi-scale features fusion method will re-learn the fusion feature through subsequent convolutional layers and gain more gait details and semantic information, as well as alleviate gradient vanishing problem during the training.

**Figure 5:**
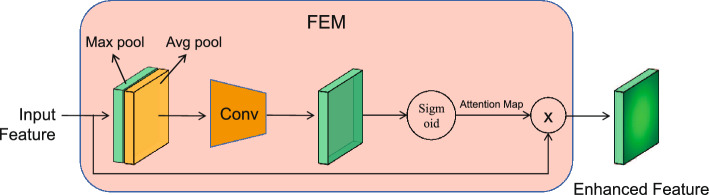
FEM module.

The FEM (Feature Enhancement Module) module composed of an attention mechanism is added to GaitSet, as shown in Fig. 5. FEM enables enhancing the input gait feature and transfer the enhanced results to subsequent modules, improving the disparity in gait between pedestrians exhibiting difference and variations within the same pedestrian.

## Experiments and results

### System experimental site selection

Considering factors like: the closed-loop characteristic of the data collected, the collective data size, and the distance between the research office and experiment spot, the line of BeijingBei-Zhangjiakou high-speed railway were chosen for experiment. We did an investigation regarding passenger flow on a day and the results could be seen in Table 2. There are the largest number of passengers between Qinghe railway station and Badaling Great Wall railway station, resulting in these two stations to be our selection. We also found that Qinghe railway station can be initial station for some trains while Badaling Great Wall railway station could only be middle stations for all trains. To keep the collected data clean, we selected Qinghe railway station as entry station (check-in) and Badaling Great Wall railway station as exit station (check-out) for experiment.

**Table 2:** Passenger OD flow in Beijing–Zhangjiakou high-speed railway in one day.

Exit									
Entry	Beijingbei	Qinghe	Changping	Badaling Great Wall	Donghuayuanbei	Huailai	Xiahuayuanbei	Xuanhuabei	Zhangjiakou
Beijingbei	–	79	26	566	19	73	125	58	1483
Qinghe	100	–	38	3909	129	754	293	1094	2972
Changping	1	107	–	0	1	0	0	8	9
Badaling Great Wall	1608	3240	1	–	17	17	6	32	73
Donghuayuanbei	0	204	0	0	–	2	3	14	30
Huailai	180	891	8	19	1	–	10	48	243
Xiahuayuanbei	32	448	3	7	1	4	–	13	98
Xuanhuabei	178	1314	19	26	8	23	17	–	178
Zhangjiakou	2023	2903	28	58	17	161	90	138	–

We also need to select the specific train for experiment at the same time. We queried all trains routing from Qinghe to Badaling to filter our experimental train and partial results could be seen in Table 3. With a long arrival time internal between different trains, the cleanliness of the collected data can be ensured to a certain extent. So the train code D6705 was chosen for experiment on contactless identification analysis.

**Table 3:** Parial train code information from Qinghe Station to Badaling Station.

Train code	Initial station	Arrival station	Arrive time	Passengers number
D1091	Qinghe	Badaling Greal Wall	10:34	662
D6705	Qinghe	Badaling Greal Wall	11:20	1174
D6712	Qinghe	Badaling Greal Wall	12:17	605

### Data collection and experimental results

**Figure 6:**
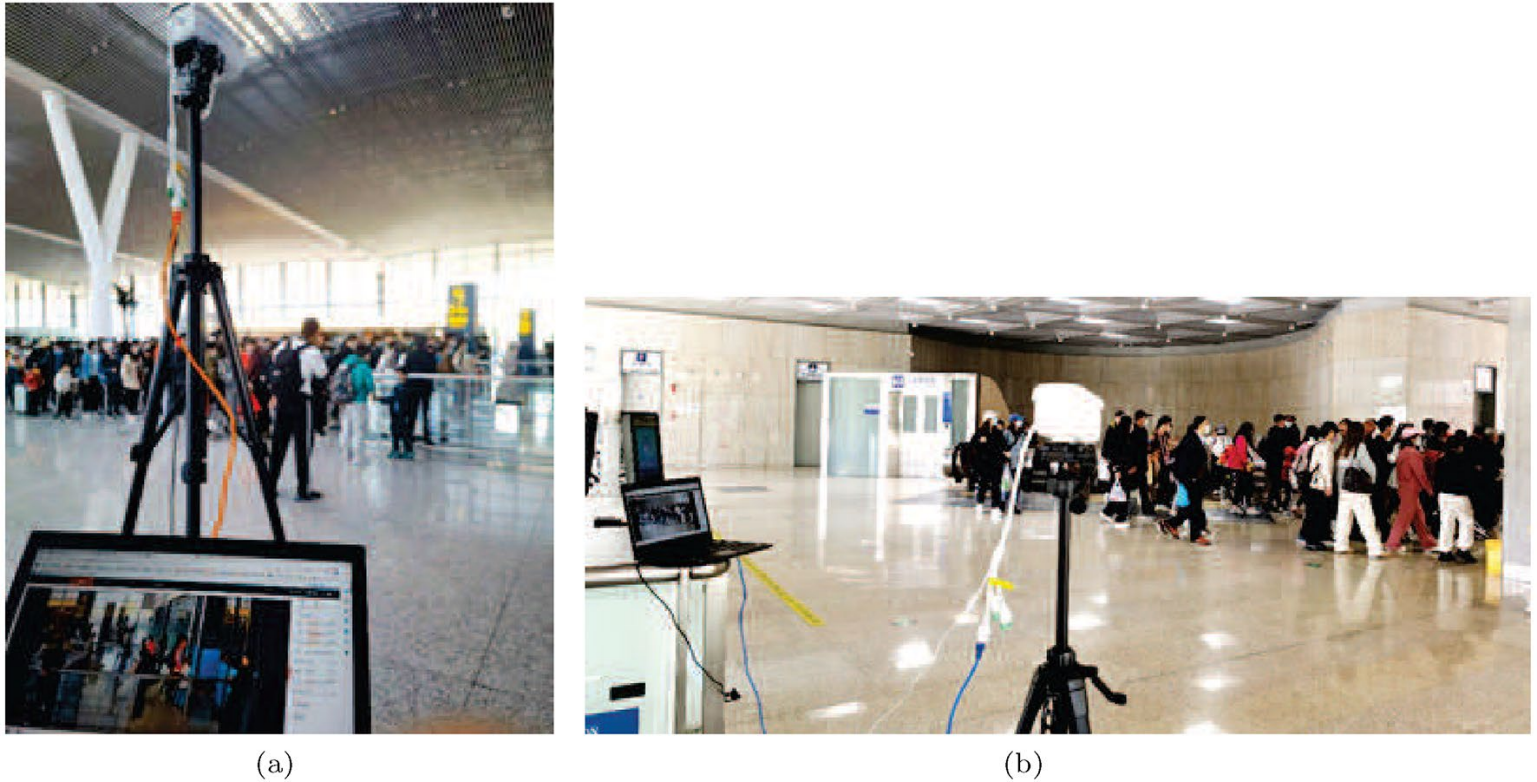
On-site camera deployment for gait. (a) Camera deployment for check-in gait. (b) Camera deployment for checkout gait.

There are 4 check-in gates for train code D6705 at Qinghe station and 7 checkout gates at Badaling Great Wall station. Each gate owns a visual panel with an embedded camera, which is used to collect passengers’ faces, and an additional camera is deployed to track and collect passengers’ gait, as shown in Fig. 6.

The camera used for gait collection has a more than 10 meters field depth. Constrained by the on-site conditions, only shot gait data between the gate and station platform could be collected for check-in passengers, which can meet the requirements of gait recognition. For checkout passengers, the longer distance gait data of whom could be collected.

### Comparative analysis of proposed Dwsegnet

For the training of the Dwsegnet segmentation algorithm, all samples labeled as human in the COCO dataset and VOC dataset are utilized to optimize the MobilenetV2 network to obtain deep features and related classifiers. We transmit the corresponding network topology and parameters as the Dwsegnet encoder, while retaining some feature maps. Only the parameters in the decoder and refinement module will be altered during the second training stage, which uses the data with weakly annotated labels as the training input. Dwsegnet’s encoder parameters will remain fixed.

**Figure 7:**
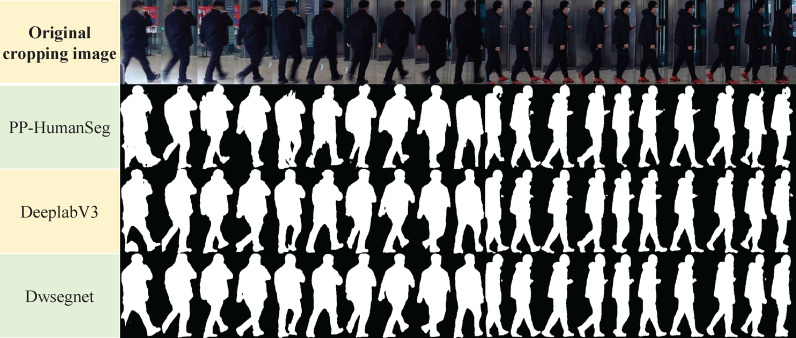
Different segmentation results varied from algorithm models.

**Figure 8:**
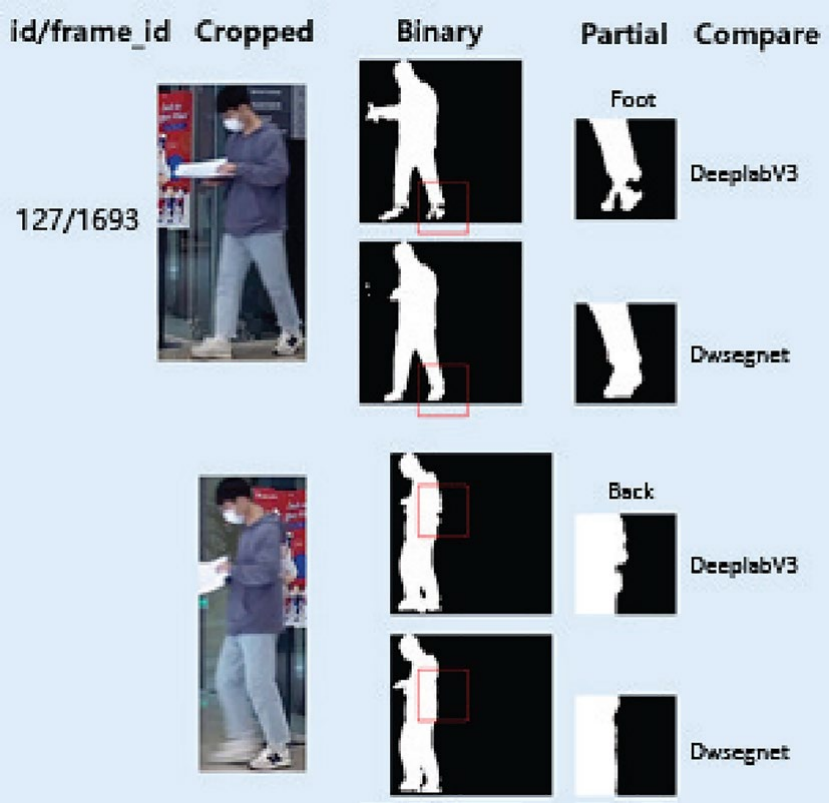
Detailed comparison with DeeplabV3 and Dwsegnet.

We perform our Dwsegnet training on four NVIDIA V100 GPUs, Adam works for optimizer of objective functions with initial learning rate $lr=1e-4$, and momentums $\beta \epsilon {\{0.9,0.999\}}$. After 300 epochs of model training, the learning rate reduces to 5e−7, and the loss value reduces from 0.09 to 0.014. The results compared with other commonly used segmentation models are shown in Figs. 7 and 8.

As indicated in Fig. 7, segmentation results from DeepLabV3 and Dwsegnet are significantly better in details than that of PP-HumanSeg. In a more refined comparison in Fig. 8, the body edges predicted by Dwsegnet are more complete and smoother than DeepLabV3’s, indicating the effectiveness of using weakly annotated labels for transfer learning.

### Ablation study with improved GaitSet

In the pretraining of the improved GaitSet, the input size is 30 $\times$ 1 $\times$ 64 $\times$ 44, where 30 is the number of input gait sequences, 1 is the number of channels, 64 is the height of a single gait image, and 44 is the width of a gait image. Adam is selected as the optimizer. The learning rate is $lr=1e-4$, and a batch size of 8 $\times$ 16 is sampled, where 8 denotes the number of persons and 16 denotes the number of training samples for each person in the batch. The triplet loss used in original GaitSet is also adopted. With 4 Nvidia GTX 2080Ti GPUs, we spend 80,000 epochs on CASIA-B dataset for pretraining. In the fine-tuning stage, we re-sets $lr=1e-4$ and execute 2000 epochs on Railway Enter Natural Gait (RENG) dataset, which is collected in real railway scenes, and GREW gait set. All the ratio of training samples to testing samples is 2:1. To verify the effectiveness of the improved GaitSet, the ablation experiment of gait recognition accuracy had been conducted on the CASIA-B, GREW, and RENG datasets, and the results are shown in Table 4.

**Table 4:** Algorithm ablation experiment on different gait datasets.

Algorithm	CASIA-B	GREW	RENG
GaitSet	95.0%	46.3%	78.39%
Proposed (Only multi-feature fusion)	95.34%	47.8%	79.40%
Proposed (Only FEM)	95.45%	47.6%	78.89%
Proposed	95.81%	48.2%	79.90%

From Table 4, some conclusions can be draw as follows: (1) On all gait datasets, the proposed model performs better than that of the proposed model with partial mudule. (2) Assembling single module of the multi-scale features fusion or FEM, the proposed model outperforms the baseline GaitSet, demonstrating the effectiveness of the proposed modules.

### The contactless checkout effectiveness of gait augmented face recognition

#### Passenger detection and image cropping

As a variant of YOLOv5, Yolov5m not only can ensure the accuracy of object detection, but also optimizes the model size and inference speed. So this paper selects Yolov5m to extract frames from videos to detect pedestrian. Each frame will be detected with the result of coordinates of detection box and identity label. According to the coordinates of detection box, the original frame is cropped to contain body parts only, and the re-identification algorithm is employed to classify cropped images of the same pedestrian into the identical directory.

#### Passenger image segmentation

**Figure 9:**
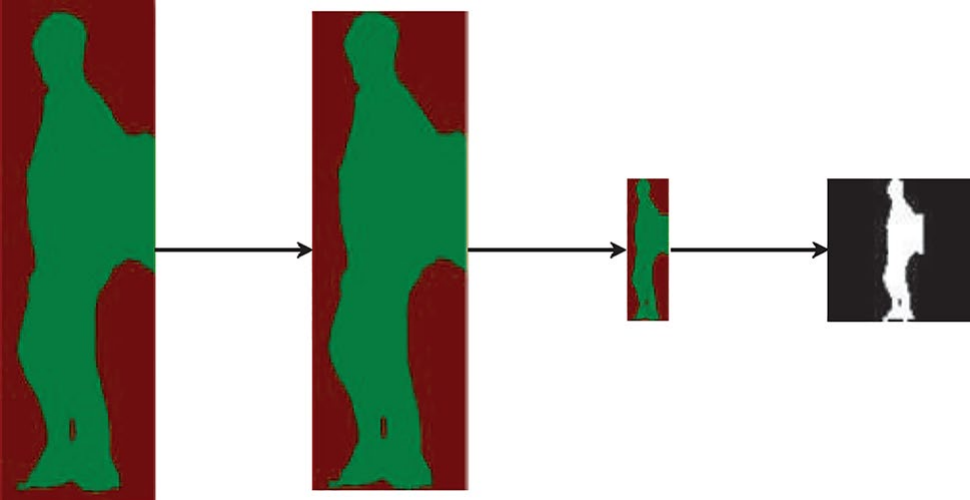
Scale standardization of binary image.

In this paper, the segmentation network of Dwsegnet is exploited to process the multi-scale cropped images and output binary images. To better support subsequent gait recognition, the segmented binary image is required to conduct scale standardization and set encapsulating. The process of scale standardization for binary images is as follows: (1) Determine the head and foot of the passenger in the binary image and execute further cropping process by the way of calculating the summation of pixel values in each row. If the summarized value is zero and the row number is as big as possible at the top, then the top of the cropping image is determined, while if the sum value is zero and the row number is as small as possible at the bottom, the bottom of the cropping image is also determined. (2) Scale the previous cutting image proportionally to a height of 64 pixels. (3) Accumulate the sum of all pixel values as the total value. (4) Accumulate the summation of pixel values from the left column to the right column as the accumulated column value. If the accumulated column value is greater than or equal to half of the total value, the current column is selected as the centerline. (5) Based on the centerline, expand left and right by 32 pixels and fill zero if the region is beyond the cropped image. Through the process above, a 64 $\times$ 64 binary image with a unified size will be obtained. The process is generally shown in Fig. 9.

**Figure 10:**
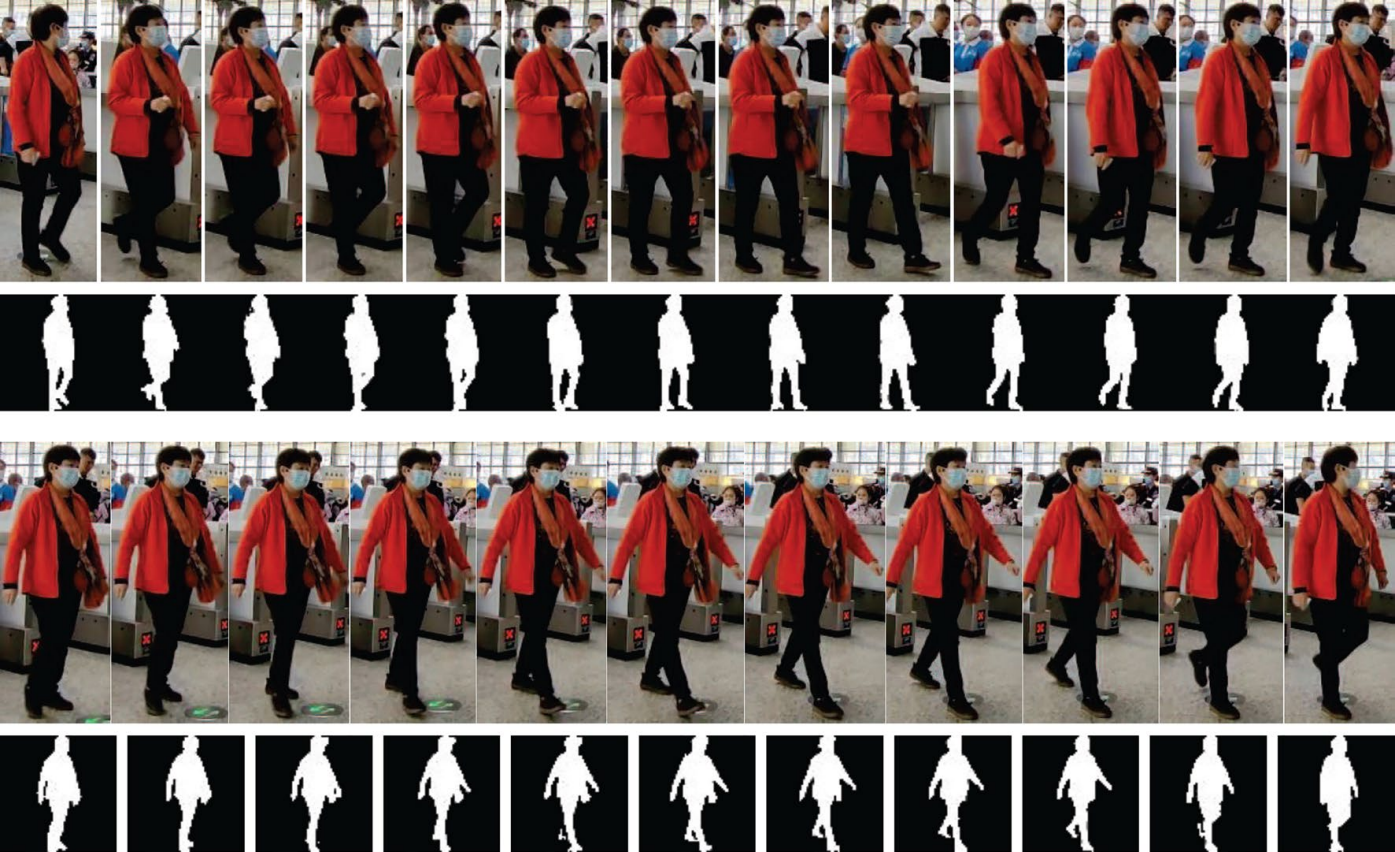
Cropping images corresponding with normalized binary image.

To extract gait features, each passenger’s binary images are encapsulated as an unordered set. A near-complete gait cycle is displayed in the image segmentation of cropped images and associated binary images with the normalized scale, as seen in Fig. 10.

#### Feature extraction and gait retrieval

The output of the HPM module is employed as the gait feature in the network model of the improved GaitSet. When passengers enter the station, their real-name information and gait features will be combined to create a pickle file and form gait gallery. When a passenger exits the station, through the similar series processing, the gait feature will be extracted as the gait probe. Following the similarity calculating with sorted scores between the probe and gallery, the gait retrieval process is finished if the top-1 similarity score exceeds the preset threshold and the real-name information of the passenger in the gait gallery is fetched. After that, subsequent processes like ticket checking and gate door opening will proceed.

#### Times and speed

We conducted a comparison of inference time across various baselines, encompassing pre-processing, gait feature extraction, and gait retrieval. Times are roughly measured by original GaitSet on the GREW test set, while GaitGL^42^ and the improved GaitSet were evaluated on our environmental data, with averaged durations of all sequences. Furthermore, we integrated a practical face algorithm to assess the time required for opening entry/exit gates in alignment with the advancements in gait recognition algorithms. As illustrated in Table 5, for an sequence averaging 157 frames, pre-processing (including detection, segmentation, and pose estimation) consumes the majority of the time, demonstrating that current gait recognition pipelines are unable to apply in real-world applications. Gait feature extraction (inference by the main network) and the retrieval process exhibit relatively faster speeds. Additionally, we computed the FLOPs and parameters of the gait networks for comparison purposes. Notably, due to the single-image requirement for face recognition, the reported time for the practical face algorithm in Table 5 represents the average duration for processing an image.

**Table 5:** Inference time, FLOPs and parameters of different algorithms.

Algorithm	Pre-process	Feature	Retrieve	Total	FLOPs	Params
Original GaitSet	45.62s	2.89s	0.00058s	48.51s	1.06G	6.31M
Improved GaitSet	48.80s	3.09s	0.00060s	51.80s	1.14G	6.89M
GaiGL	53.59s	0.25s	0.00041s	53.84s	0.76G	3.10M
Practical face	0.052s	0.15s	0.1s	0.302s	2.95G	122.53M

#### Analysis of fusion data

Apart from speed, accuracy also plays a critical role in practical gait recognition. During the experiment, on a particular day in April 2023, there are 569 passengers on train D6705, who depart from Qinghe station to Badaling Great Wall station and Yanqing station. Of them, 519 passengers are arriving at Badaling Great Wall station and 50 passengers are arriving at subsequent Yanqing station. The size of the constructed face gallery is 509 due to the lack of a certain passengers’ facial features, while the size of the constructed gait gallery is 452 due to the filtering of gait collection. Passengers who arrive at subsequent Yanqing station will be included in the gait gallery, because it is unable to distinguish passengers who will arrive at Badaling Great Wall station or Yanqing station. The passenger’s real-name information is linked to both the face and gait galleries.

As shown in Table 6, when passengers exit Badaling Great Wall station, 357 passengers are successfully identified by face retrieval, and only 23 passengers are successfully identified by gait retrieval through improved GaitSet, of which 12 passengers are identified via the proposed gait-augmented face recognition process, improving the contactless checkout rate by 2.31%.

**Table 6:** Statistical analysis of the contactless checkout rate (Improved GaitSet).

–	Face	Gait	Crossed passengers number	Gait-assisted improvement
Qinghe entering	519 + 50	519 + 50	–	–
Badaling great wall exiting	519	519	–	–
Gallery size	509	452	–	–
Retrieval success number	357	23	11	12
Retrieval success rate	$\frac{357}{509}=70.14\%$	$\frac{23}{452}=5.09\%$	–	$\frac{12}{452}=2.65\%$
Exit success rate	$\frac{357}{519}=68.79\%$	$\frac{23}{519}=4.43\%$	–	$\frac{12}{519}=2.31\%$

To our surprise, the outcome indicates that gait recognition may not perform optimally to empower our production system. In order to augment a high-accuracy gait-recognition-assisted research, a transverse comparison work is conducted with another well-known gait recognition algorithm, GaitGL, which performs well on public outdoor databases and is popular in industry communities. GaitGL integrates the spatially global gait representations with the local region-based descriptors and is implemented on the PyTorch-based OpenGait codebase, utilizing a structurally simple and experimentally powerful GaitBase baseline model. We anticipate gaining higher accuracy by incorporating GaitGL to analyze our real-world railway station data. The experiment result with GaitGL is exhibited in Table 7.

**Table 7:** Statistical analysis of the contactless checkout rate (GaitGL).

–	Face	Gait	Crossed passengers number	Gait-assisted improvement
Qinghe entering	519 + 50	519 + 50	–	–
Badaling great wall exiting	519	519	–	–
Gallery size	509	452	–	–
Retrieval success number	357	28	12	13
Retrieval success rate	$\frac{357}{509}=70.14\%$	$\frac{28}{452}=6.19\%$	–	$\frac{13}{452}=2.87\%$
Exit success rate	$\frac{357}{519}=68.79\%$	$\frac{28}{519}=5.39\%$	–	$\frac{13}{519}=2.5\%$

Unfortunately, the gait recognition result with GaitGL, 6.19% retrieval success rate and 5.39% exit success rate, is still feeble, having only marginally increased when compared to the improved GaitSet approach, which dissatisfies us so much and demonstrates impossibility and vulnerability in practical usage.

In summary, although gait identification recognition enables slightly improving the contactless checkout rate for passengers in real scenarios to a certain extent, too low success rate and too high reference time is unable to meet our expectation. Through further analysis, in real scenarios, the technique of gait recognition would encounter fully-unconstrained challenges and troublesome real-world noisy factors, such as diverse view, occlusion, various carrying and dressing, complex and dynamic background clutters, illumination, walking style, surface influence et al. With regard to our research, reasons are mainly as follows: (1) The extraction of gait feature is impeded by severe crowed occlusion among passengers in real railway station environment. (2) The manner how passengers backpack, pull boxes, or carry children, differs greatly with their check-in and check-out in railway station, which makes a significant impact on passengers’ silhouettes resulting in efficacy loss of gait recognition algorithms. (3) Since a tiny proportion of passengers will enter station through manual gate as opposed to our data-auto-collected gate, gait information are missing for these passengers in the gait gallery. (4) The gait recognition algorithm’s capacity is still inadequate when confronted with genuine fully-unconstrained circumstances. The aforementioned noise elements offer a crucial foundation for in-depth study of forthcoming practical applications.

### Statement attesting

Informed consent was obtained from all subjects for publication of identifying information/images in an online open-access publication.

## Conclusion

This paper presents a contactless passenger checkout process utilizing gait-augmented face recognition, where face recognition serves as the primary method and gait recognition as a supplementary technique. The integrated system introduces key technologies, including the Dwsegnet body segmentation algorithm and an enhanced GaitSet algorithm for gait recognition. To validate this contactless checkout process and the key technologies, gait data from check-in passengers at Qinghe station is utilized as gait gallery, while gait data from check-out passengers at Badaling Great Wall station is collected as gait probe. Through benchmarking, the gait-augmented face recognition process can enhance the identification rate for checkout passengers, confirming the efficacy with the proposed approach and corresponding components. Despite of the modest improvement, real-world railway station complexities limit the extent of enhancement. If it were to be used in the future, gait recognition algorithms have to be optimized for real-time production environments, and station gate processing capability and hardware integration have to be continually enhanced.

## Data Availability

The datasets generated and analyzed during the current study are not publicly available due to the privacy protection required by China Railway Group but are available from the corresponding author on reasonable request.
